# The potentially therapeutic effects of ascorbic acid in different cell line in attempt to reduce the risk of radiation therapy

**DOI:** 10.1038/s41598-025-96697-x

**Published:** 2025-04-29

**Authors:** Rasha S. Shams El Dine, Heba T. Youseef, Ashraf K. Awaad, Sabahh I. Hammoury, Ehab I. Mohamed

**Affiliations:** 1 Medical Biophysics Department, Medical Research Institute, Alexandria University, Alexandria, Egypt; 2 Biochemistry and Molecular Biology Department, Alexandria University, Alexandria, Egypt; 3 Radiotherapy Department, Ayadi Al-Mostakbal Oncology Hospital, Alexandria, Egypt

**Keywords:** Leukemia, Ascorbic acid, X-irradiation, Autophagy, Cell proliferation, Hypoxia-inducible factor, Apoptosis, THP-1 cells, K562 cells, Oxidative stress, Biophysics, Cancer

## Abstract

Leukemia is the most common type of serious, life-threatening cancer that requires the immediate initiation of therapy. Ascorbic acid (AsA), commonly known as Vitamin C, has been gaining attention due to its antioxidant activity as a potential treatment for human malignancies. In this study, the THP-1 monocytic cell line was treated with two doses of AsA: a low dose (L-AsA, 2.5 µg/mL) and a high dose (H-AsA, 5 µg/mL), while the K562 lymphocytic cell line was treated with two doses of AsA: a low dose (L-AsA, 4 µg/mL) and a high dose (H-AsA, 8 µg/mL). After a 24-h incubation period, all cells were exposed to different doses of X-radiation (2, 4, 8 Gy). The viability of THP-1 and K562 treated by AsA was assessed using the MTT assay. Additionally, we evaluated apoptosis, autophagy, proliferation, cell cycle progression, hypoxia-inducible factor (HIF-1), malondialdehyde (MDA), and total antioxidant capacity (TAC). Our study demonstrated that AsA, in combination with X-radiation, induced significant apoptosis and notably reduced Ki67 levels in human leukemia THP-1 cells. Furthermore, X-radiation caused DNA damage, leading to cell cycle arrest at the G0/G1 phase in THP-1 cells. Moreover, AsA significantly reduced HIF-1 levels, which are essential for the survival of tumor cells in hypoxic conditions. We also found that the administration of AsA in combination with X-radiation had a synergistic and dose-dependent effect on THP-1 and K562 cells. Notably, the combination of L-AsA with 2 Gy X-radiation showed a more pronounced effect than 8 Gy X-radiation alone. These results suggest that AsA has promising anti-proliferative, pro-apoptotic, and autophagic effects on leukemic cells. Furthermore, the dose of X-radiation may be reduced when combined with AsA in an effort to minimize its potential side effects.

## Introduction

Leukemia is a malignant tumor of the hematological system, commonly classified as a neoplasm. It causes significant impairment to human health and is a major cause of cancer-related fatalities worldwide^1^. Leukemia represents approximately 5% of all cancer cases and ranks sixth among various human malignancies^2^. The exact etiology of leukemia remains unknown^3^. After initial induction chemotherapy, patients receive risk-appropriate post-remission consolidative treatments. Although most acute myeloid leukemia (AML) patients respond to initial chemotherapy, refractory cases are common, and recurrence remains a significant challenge^4^. Many cancer patients use complementary and alternative therapies, including nutritional supplements like antioxidants such as ascorbic acid (AsA)^5^. AsA is a fundamental low-molecular-weight antioxidant present in the human body. It is an essential nutrient with various positive effects and exists in the body in the form of ascorbat^6^. Leukemia often depletes AsA levels to very low concentrations, and any remaining AsA in the bloodstream is scavenged and sequestered by leukocytes^7^. Pauling and Cameron first proposed high-dose AsA as a potential anticancer agent in the 1970s^8^. Recent preclinical studies have validated AsA’s anticancer efficacy and selective cytotoxicity in numerous human malignancies, both in vitro and in vivo^9^. Research on hematological malignancies has shown that AsA exerts cytotoxic effects on leukemic cells while causing minimal damage to healthy cells^10,11^.

The primary mechanism behind AsA’s anticancer effect involves pro-oxidant damage through auto-oxidation, leading to the formation of reactive oxygen species (ROS)^12,13^. High ROS levels are cytotoxic, causing DNA and mitochondrial damage and triggering apoptotic pathways^13^.

AsA may also reduce the incidence of secondary cancers in normal cells treated with radiation therapy^14^.

Radiotherapy offers significant advantages over chemotherapy by delivering localized ionizing radiation to tumor tissues while sparing normal tissues^15^. Currently, radiation therapy is used for approximately 50% of cancer patients^16^ and contributes to about 40% of cancer cures^17^. The effectiveness of X-radiation is often associated with increased ROS production and apoptosis^18^. High-energy X-radiation can kill cancer cells by directly or indirectly damaging DNA or by inducing ROS to degrade DNA^19^. ROS levels must be regulated by a robust natural antioxidant system. In hematological cancers, disruption of this balance leads to oxidative stress and increased ROS interactions with endogenous cellular components^20^.

Tumor progression is linked to hypoxia, which necessitates adaptive mechanisms for survival under low oxygen conditions^21^. Hypoxia-inducible factor 1 (HIF-1) consists of two subunits, HIF-1α and HIF-1β, that regulate genes involved in metabolic reprogramming, including angiogenesis, anti-apoptotic mechanisms, stem cell renewal, invasion, and metastasis, and resistance to therapy^22^. HIF transcription factors play a crucial role in cancer progression and modify cellular responses to ionizing radiation in hypoxic microenvironments^23^. HIF-1 is a potential target for enhancing radiotherapy outcomes^24^. Additionally, AsA-deficient cells may exhibit increased HIF-1α activity, potentially contributing to tumor formation. Ascorbate therapy stimulates HIF hydroxylase activity, reducing HIF-1α levels and inhibiting tumor growth. High-dose AsA therapy has been shown to suppress tumor development by modulating HIF-1α^25^.

Autophagy, or self-eating, is an evolutionarily conserved process where cells degrade proteins and damage organelles to maintain homeostasis^26^. Autophagy responds to various stressors, including organelle damage, nutrient deprivation, protein aggregation, hypoxia, and ionizing radiation. Although autophagy is typically protective, excessive autophagic activity can lead to cell death^26^. Physiological and elevated levels of ascorbate increase the expression of LC3B, suggesting a potential role of autophagy in ascorbate-mediated cancer cell death^27^.

## Materials and methods

### Cell lines and reagents

THP-1 monocytic and K562 lymphoblastic cell lines were obtained from the Center of Excellence Egypt. All the cells were maintained in RPMI-1640 (Sigma-Aldrich Corporation, St. Louis, Missouri, USA), supplemented with 10% fetal bovine serum (Biowest, Nuaillé, France), 100 U/mL penicillin (Life Technologies, Carlsbad, CA) and 100 μg/mL streptomycin (Life Technologies, Carlsbad, CA) in a humidified atmosphere containing 5% CO_2_ at 37 °C. Vitamin C (Ascorbic Acid) was purchased from Sigma Aldrich (Merck Millipore, Darmstadt, Germany).

### Ascorbic acid

AsA was obtained from El Gomhorya company (520 plastic container X 100gm), white crystalline, optical rotation 20.5*, heavy metals ≤ 10 ppm, oxalic acid ≤ 0.3%, sulfate ≤ 0.1% and assay (chemical) 99–100.5%.

### X-radiation

The linear accelerator (Siemens Primus 2) is a model device that can perform therapies with 6 mV X-radiation at Alexandria Al Mostakbal Oncology Hospital, Egypt for all the cells.

Exponentially growing THP-1 cells were suspended with a complete medium in a 6-well plate (5 × 10^5^/well). THP-1 and K562 cells were irradiated under the following conditions, field size 20*20 cm, SSD 95 cm, field array anterior and posterior, with a dose rate of 300 monitor units/min for doses 2 & 4 & 8 Gy at room temperature.

### Cell viability assay

The viability test was measured by 3-(4,5-Dimethylthiazol-2-yl)-2,5-Diphenyltetrazolium Bromide (MTT). 5,000 THP-1 and K562 cells/well were seeded for each cell line in 96-well plates. AsA was added to the cultured THP-1 and K562 at concentrations 0.125, 0.25, 0.5, 1, 2, 4, 8, 16 and 32 µg/mL. After 24 h, MTT reagent (Sigma-Aldrich; Merck Millipore) was applied to each well and incubated for 4 h at 37 °C. The cell supernatant was discarded, and the formazan precipitates were dissolved in 100 µl dimethyl sulfoxide (DMSO). An optical density was measured at 570 nm, and a growth curve was constructed^28^. The same concentrations of AsA, used previously with THP-1 and K562, then MTT reagent was applied for evaluation of the cytotoxic effect of AsA.

Monocytes were isolated from the blood according to the protocol conducted by Lara, T., E. et al.^29^.

### Determination of IC50 and combination index

The inhibitory concentration of AsA (IC50) was determined from the dose–response curve utilizing Compusyn software (CombuSyn, Inc., Paramus, USA). The efficacy of the combined treatment modalities X-irradiation and AsA was evaluated through the calculation of the combination index (CI) using the same software.

The combination index for a two-drug combination can be calculated as following:

$$CI = \frac{{(D)_{1} }}{{(Dx)_{1} }} + \frac{{(D)_{2} }}{{(Dx)_{2} }}$$

where (Dx)_1_ represents the dose of the drug D_1_ alone that inhibits the growth of cells by x% and (Dx)_2_ is the dose of the drug D2 alone that inhibits the growth of cells by x%.

It has its graphical representation consisting of a plot of CI versus effect, where CI < 1, CI = 1, and CI > 1 indicate synergism, additivity, and antagonism, respectively^30^. The IC50 for AsA after a 24-h treatment was determined to be 5 μg/mL for THP-1 cells and 8 μg/mL for K562 cells as calculated by Compusyn software

Based on MTT assay results and Compusyn software, AsA concentrations of 5 μg/mL and 2.5 μg/mL for THP-1 cells and 8 μg/mL and 4 μg/mL, for K562 cells were selected for further experimentation as low and high AsA doses.

### Experimental design

According to MTT assay results, AsA significantly reduced the number of viable THP-1 and K562 cells in a concentration-dependent manner, with the IC50 value for these cell lines.

THP-1 and K562 tumor cells were seeded in 6-well plates (3 × 10^5^/well), and then were divided into three main groups each group was further subdivided into 4 subgroups:

Control group: The cultures of this group were irradiated with various doses of X-radiation:

no radiation (Ctrl_0Gy_), 2 Gy X-radiated (Ctrl_2Gy_) 4 Gy X-radiated (Ctrl_4Gy_), and 8 Gy X-radiated (Ctrl_8Gy_).

- low dose of AsA group: The cells of this group were treated with low concentration (half of IC50, 2.5 μg/ml for THP-1 cells and 4 μg/ml for K562 cells) of AsA for 24 h before exposure to X-radiation: 0 Gy (L-AsA_0Gy_), 2 Gy (L-AsA_2Gy_),4 Gy (L-AsA_4Gy_) and 8 Gy (L-AsA_8Gy_).
- High dose of AsA group: The cells of this group were treated with high concentration (IC50, 5 μg/ml for THP-1 cells and 8 μg/ml for K562 cells) of AsA for 24 h before exposure to X-radiation: 0 Gy (H-AsA_0Gy_), 2 Gy (HAsA_2Gy_), 4 Gy (H-AsA_4Gy_) and 8 Gy (H-AsA_8Gy_).

All cultures were harvested after 24 h post-irradiation.

### Apoptosis assay

To assess the apoptosis rate of cells, THP-1 and K562 tumor cells were stained with an annexin-V-Alexa Fluor 488 and propidium iodide (PI) kit (BD Biosciences Pharmingen TM, Becton Dickinson, USA), according to the manufacturer’s protocol. Apoptosis was detected by flow cytometry (BD FACS Calibur, San Jose, CA, USA) at an excitation wavelength of 488 nm and emission wavelengths of 525 ± 20 nm (Annexin-V) and 585 ± 20 nm (PI).The data were analyzed using BD Cell Quest Pro Software, version 5.1 (BD Biosciences, San Jose, CA, USA)^31^. The results are expressed as the percentage of viable, early apoptotic, late apoptotic/necrotic, and necrotic cells. All experiments were repeated three times.

### Colony formation assay

The soft agar colony formation assay is a standard technique used to evaluate the ability of cells to proliferate without attachment, a characteristic indicative of tumorigenic potential. Colony formation assay was conducted according to Borowicz et al.^32^ Briefly, 6-well plates were first prepared by coating each well with 1.5 mL of 0.6% noble agar in a complete RPMI medium. The agar was allowed to solidify at room temperature for 30 min. Subsequently, cells were treated with AsA and/or X-radiation for 24 h, using different concentrations of AsA (2.5 and 5 μg/mL) and radiation doses (2&4&8Gy) as an experimental variable.

After treatment, 1000 cells from each group were mixed with complete media containing 0.3% noble agar (0.5 mL/well). This cell-agar mixture was layered over the pre-solidified base agar and allowed to solidify for an additional 30 min. To prevent desiccation during the incubation period, 200 μL of complete media was added to each well. The plates were then incubated at 37 °C with 5% CO_2_ for 21 days.

After the incubation period, the colonies that had formed were stained with 0.01% crystal violet for 1 h to enhance visualization. Colony images were captured using a microscope, and only those with a diameter greater than 50 μM were counted. The quantification of colonies was performed using the Image J software (National Institutes of Health, Bethesda, MD, USA), allowing for precise and reproducible measurement of colony formation.

### Proliferation assay

Proliferative activity was determined on THP-1 cells using a fixation and permeabilization method with methanol. Briefly, Cells were incubated with 500 µL of 95% frozen methanol for 1 h. The cells were rinsed in 1× phosphate-buffered saline (PBS) and treated with Alexa Fluor 488-conjugated anti-Ki67 antibody (Cell Signaling Technology, MA, USA) for 1 h.

After incubation, samples were washed in 1× PBS, resuspended, and analyzed immediately on a BD FACSCalibur flow cytometer. A minimum of 10,000 events were collected for Ki67 determination. The data were analyzed using the BD CellQuest Pro Software, version 5.1 (BD Biosciences, San Jose, CA, USA). Proliferative activity was quantified as a proportion of Ki67positive cells. A gate of analysis was presented on the FSC vs SSC scattergram to remove debris and background, and the proportion of positive cells was computed on SSC versus fluorescence intensity^33^.

### Cell cycle analysis

THP-1 cells were treated with two concentrations of AsA (2.5, 5 μg/ml) for 24 h, followed by exposure to X-irradiation (2 Gy, 4 Gy, 8 Gy), and then cell cycle assay was applied. After 24 h of radiation exposure, both treated and untreated cells were collected, washed twice with 1× PBS, and fixed with 95% methanol (pre-cooled) with gentle vortexing for 4 h at 4 °C. After 4 h of incubation, the fixed cells were centrifuged at 1800 rpm for 5 min to remove the methanol. Subsequently, Cells were treated with 1 mg/mL RNase A (Qiagen Hilden, Germany) and 50 µg/mL PI for 1 h at room temperature. The DNA distribution was determined using a FACSCalibur flow cytometer (BD Biosciences).

The cells were washed twice with 1× PBS, resuspended in 500 mL of 1× PBS with 0.1 mg/mL Ribonuclease A, and incubated at room temperature for 30 min. After incubation, 0.01 mg/mL of PI was given to each tube, allowing the fluorescence of the PI-DNA complex to be detected at various phases of the cell cycle using Flow Cytometry BD CellQuest Pro Software, version 5^34^. The results are given as the proportion of cells in each phase of the cell cycle (G0/G1, S, and G2/M) based on DNA content histograms.

### HIF-1α determination

HIF-1α expression was measured by flow cytometry. THP-1 cells were harvested, fixed with 4% paraformaldehyde in PBS for 30 min at 4 °C, permeabilized with 0.1% Tween-20 in PBS for 10 min at 4 °C, and stained with anti-HIF-1α (dilution, 1:50; R&D Systems, Inc., Minneapolis, MN, USA) phycoerythrin (PE)-conjugated antibodies for 30 min at 4 °C. Data were taken using a BD FACSCalibur flow cytometer equipped with a 488 nm laser for excitation and a 574/26 BP filter to gather fluorescence emission. THP-1 cells were gated by physical criteria, and cell aggregates were excluded from the investigation^35^.

### Immunocytochemistry

After treatment, THP-1 washed off with PBS, fixed with 4% paraformaldehyde, permeabilized with 0.1% Triton X-100, and stained with anti-LC3B antibody at 1:250 dilution (ab225383, Abcam, Cambridge, MA, USA) for 1 h at room temperature. The LC3B antibody was visualized and acquired using a Leica TCS SP8 confocal microscope (Leica Microsystems, Weltzar, Germany) with Leica-LasX software (V4). The photos were collected at 63× magnification and analyzed with Fiji New ImageJ^36^.

### Evaluation of oxidative stress markers index (MDA/TAC index)

Oxidative stress status was indirectly assessed by measuring the ratio of the lipid peroxidation product malondialdehyde (MDA) to total antioxidant capacity (TAC) (MDA/TAC oxidative stress index) in the culture media at different AsA concentrations and different doses of X-irradiation. MDA was measured using the thiobarbituric acid (TBA) spectrophotometric method^37^ based on the reaction of MDA and TBA under high temperature, forming a colored adduct measured at 534 nm. TAC was measured by calorimetry based on the reaction of antioxidants in the sample with exogenously provided hydrogen peroxide *H*_2_*O*_2_, with the generation of a chromophore measured at 505 nm^38^. Oxidative stress index (MDA/TAC) ratio was calculated by dividing the value of oxidized molecule (MDA) by antioxidant (TAC) after converting the units of TAC to nmol/ml for each reading. If the redox balance shifted toward the oxidative side, this would represent oxidative stress and vice versa^39^.

### Statistical analysis

Data are expressed as mean ± standard deviation. The IC50 values were calculated from cell viability assay data using CompuSyn software. To assess statistical significance among groups, one-way ANOVA was employed, followed by post-hoc analysis via the Tukey test, using GraphPad Prism version 4.0 (GraphPad Software Inc., San Diego, California). All experiments were conducted in triplicate, and a p-value of < 0.05 was considered statistically significant.

## Results

The effects of radiation therapy and AsA, both separately and in combination, on THP-1 cells, were evaluated using assays for apoptosis, proliferation, cell cycle distribution, autophagy, hypoxia-inducible factor-1 (HIF-1), malondialdehyde (MDA), and total antioxidant capacity (TAC).

### Effects of AsA on THP-1, K562, and normal monocyte cell viability

**Fig. 1:**
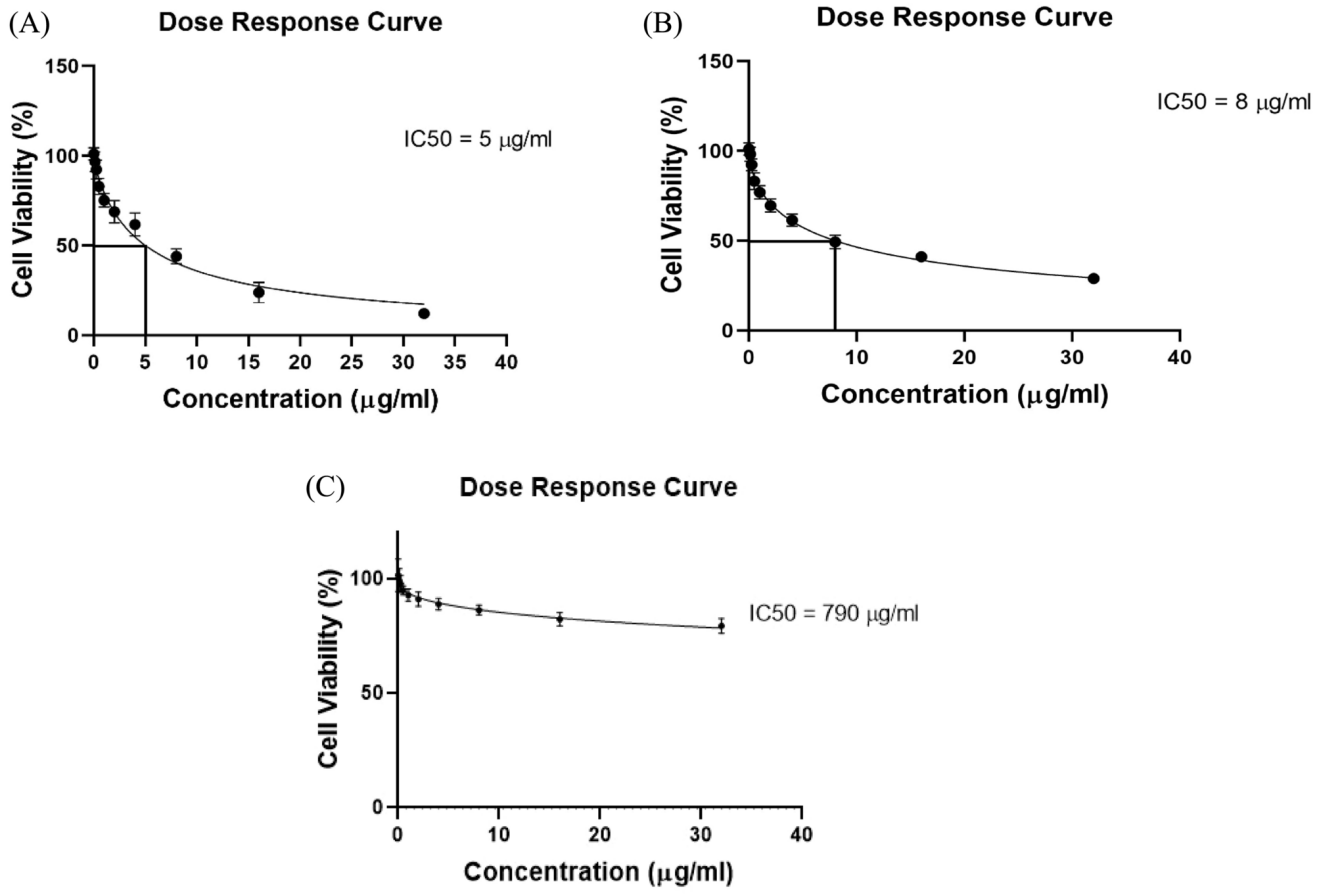
(**A**) The reduction in THP-1 cell viability was observed in response to increasing concentrations of AsA. The dose–response curve for THP-1 cells was generated following a 24-h treatment with different concentrations of AsA (0.125, 0.25, 0.5, 1, 2, 4, 8, 16 and 32 µg/mL). (**B**) The decrease in the viability of K562 cells was observed with increasing concentrations of AsA. (**C**) The dose–response curve of normal monocytic blood cells. The dose–response curve for normal monocytic cells was generated following a 24-h treatment with different concentrations of AsA (0.125, 0.25, 0.5, 1, 2, 4, 8, 16, and 32 µg/mL).

Cell viability was assessed using the MTT assay. THP-1, K562 and normal monocyte cells were exposed to a range of AsA concentrations (0.125, 0.25, 0.5, 1, 2, 4, 8, 16, and 32 µg/mL). AsA significantly reduced the number of viable THP-1 cells in a concentration-dependent manner, with an IC50 value of 5 µg/mL, as illustrated in Fig. 1A, while the IC50 value was 8 µg/mL for K562 cells that shown in Fig. 1B. but normal monocytic cells were exposed to varying concentrations of AsA, which acts as an antioxidant for these cells. Our results indicated that the IC50 value for THP-1 cells was found to be 5 µg/mL, while the IC50 for normal monocytic cells was considerably higher, at 790 µg/mL, these findings confirm that AsA has no cytotoxic effect for normal cells, and it is highly selective for THP-1 cancer cells (Fig. 1C). The IC50 value (with a 95% confidence interval) for all cell lines was determined using GraphPad Prism nonlinear regression analysis. The inhibitor vs. response-variable slope (four parameters) equation provided the best fit for the dose-response curve.

Furthermore, for the THP-1 cells, the IC50 for AsA after a 24-h treatment was determined to be 5 μg/mL, as calculated by Compusyn software as described in the material and methods section. Moreover, the combination index (CI) values for low concentrations of AsA combined with X- radiation at doses 2, 4, and 8 Gy were equal to 0.40 ± 0.07, 0.28 ± 0.03, and 0.24 ± 0.02, respectively. Additionally, high concentrations of AsA combined with the same X-radiation doses, CI values were equal to 0.43 ± 0.04, 0.33 ± 0.03, and 0.30 ± 0.04 (Table1).

**Table 1:** Combination index analysis of AsA and X-radiation reveals synergistic effects on THP-1 cell viability.

AsA/X-radiation doses		Effect	Combination Index (CI)	Evaluation of effect level
AsA	L-AsA (2.5 μg/ml)	0.29	–	–
	H-AsA (5 μg/ml)	0.51	–	–
X-radiation	Ctrl 2Gy	0.19	–	–
	Ctrl 4G	0.42	–	–
	Ctrl 8G	0.6	–	–
AsA/X-radiation	L-AsA 2 Gy	0.72	0.43	Synergy
	L-AsA 4 Gy	0.87	0.30	Synergy
	L-AsA 8 Gy	0.94	0.25	Synergy
	H-AsA 2 Gy	0.82	0.45	Synergy
	H-AsA 4 Gy	0.89	0.37	Synergy
	H-AsA 8 Gy	0.92	0.40	Synergy

While in K562 cells, IC50 for AsA was 8 μg/mL. In addition, CI values for low concentrations of AsA combined with X-radiation at doses 2, 4, and 8 Gy were equal to 0.44 ± 0.11, 0.39 ± 0.07, 0.41 ± 0.09, respectively. Furthermore, with high concentrations of AsA combined with the same X-radiation doses, CI values were equal to 0.34 ± 0.12, 0.18 ± 0.08, and 0.17 ± 0.07 (Table2).

**Table 2:** Results of the CompuSyn report for K562 cells.

Vit C/X-radiation doses		Effect	Combination Index (CI)	Evaluation of effect level
Vit C	L-AsA (4 μg/ml)	0.39	–	–
	H-AsA (8 μg/ml)	0.51	–	–
X-radiation	Ctrl 2Gy	0.21	–	–
	Ctrl 4G	0.46	–	–
	Ctrl 8G	0.68	–	–
Vit C/X-radiation	L-AsA 2 Gy	0.68	0.44	Synergy
	L-AsA 4 Gy	0.81	0.39	Synergy
	L-AsA 8 Gy	0.9	0.42	Synergy
	H-AsA 2 Gy	0.79	0.34	Synergy
	H-AsA 4 Gy	0.93	0.18	Synergy
	H-AsA 8 Gy	0.97	0.18	Synergy

**Fig. 2:**
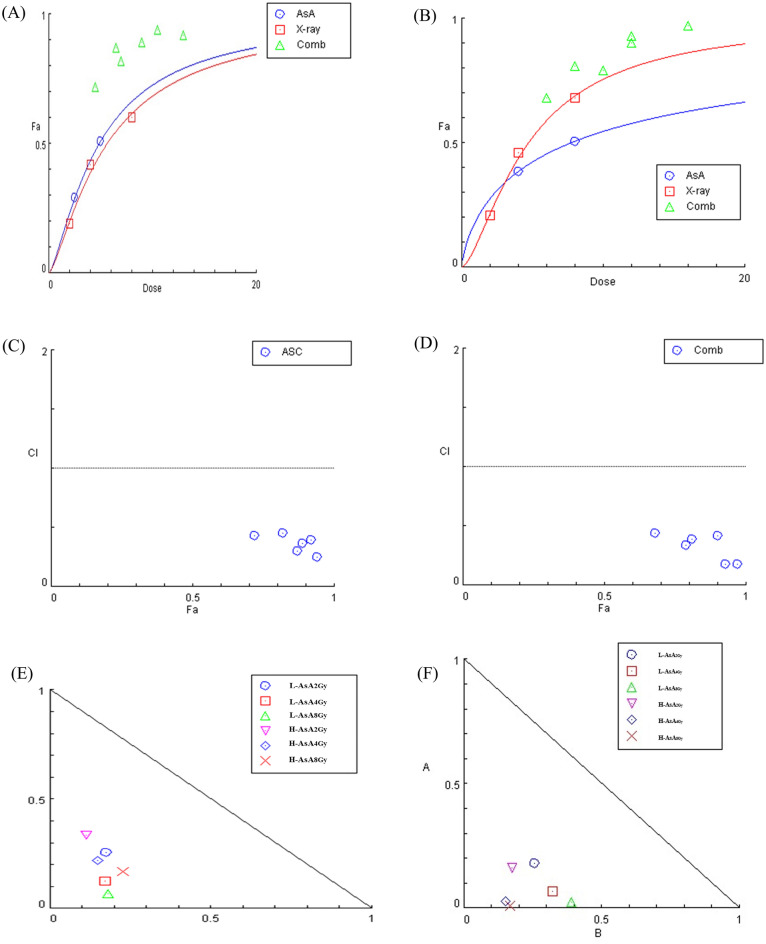
The combination index (CI) analysis which obtained from Compusyn confirms that the combination of AsA and X-radiation exhibits a synergistic effect, suggesting potential therapeutic benefits for THP-1 and K562 cell treatment. (**A**) The Dose–Effect Curve of THP1cells, the combination index (CI) values for low (2.5 µg/mL) and high (5 µg/mL) concentrations of AsA combined with X- radiation at doses 2, 4, and 8 Gy for THP-1 cells. (**B**) Dose–Effect Curve of K562 cells, specifically pertain to the CI values of K562 cells, low doses of AsA (4 µg/mL) and high (8 µg/mL) highlighting the interaction between AsA and different doses of ionizing radiation. (**C** and **D**) Combination Index (CI) plot for the interaction between AsA and X-radiation in THP-1 and K562 cells, respectively. The CI values are plotted against the fraction affected (Fa). Data points below CI = 1 indicate a synergistic effect, suggesting that the combination treatment enhances cytotoxicity compared to individual treatments. (**E** and **F**) Isobologram curve of the combination between AsA and X-radiation in leukemia cells (THP-1 and K562) respectively. The diagonal line represents the additive effect, while data points below the line indicate synergy and points above the line indicate antagonism. The observed distribution of data suggests a synergistic interaction between AsA and X-radiation, supporting their potential combined therapeutic efficacy. The strongest synergy was detected at higher radiation dose like 8 Gy which combined with AsA, suggesting enhanced therapeutic potential.

These results indicating a synergistic interaction between AsA and ionizing radiation in all cells (Fig. 2A,B).

To evaluate the interaction between AsA and X-radiation in leukemia cells, we performed a Combination Index (CI) analysis. Figure 2C,D illustrates the CI values plotted against the fraction affected (Fa), where CI < 1 indicates synergy, CI = 1 denotes an additive effect, and CI > 1 suggests antagonism. As shown in the plot, all combination treatments resulted in CI values below 1 across different Fa levels, confirming a synergistic effect between AsA and X-radiation. The most pronounced synergy was observed at higher radiation doses (8 Gy) in combination with both low and high concentrations of AsA, with CI values ranging from 0.18 to 0.45.

Moreover, the isobologram curve produced by Compusyn software was analyzed to evaluate further the interaction between AsA and varying doses of X-radiation. Points on the line of the isobologram indicate an additive effect, while points below or above the line signify synergistic or antagonistic interactions, respectively. As depicted in Fig. 2E,F, the combination of AsA and X-radiation across different doses demonstrated synergistic effects for all leukemia cell lines, with all data points situated below the line of an additive effect. These results are consistent with the CI findings, reaffirming the synergistic interaction between the treatments.

### Effect of ascorbic acid (AsA) and X-radiation on THP-1 and K562 Cells’ cellular apoptosis

**Fig. 3:**
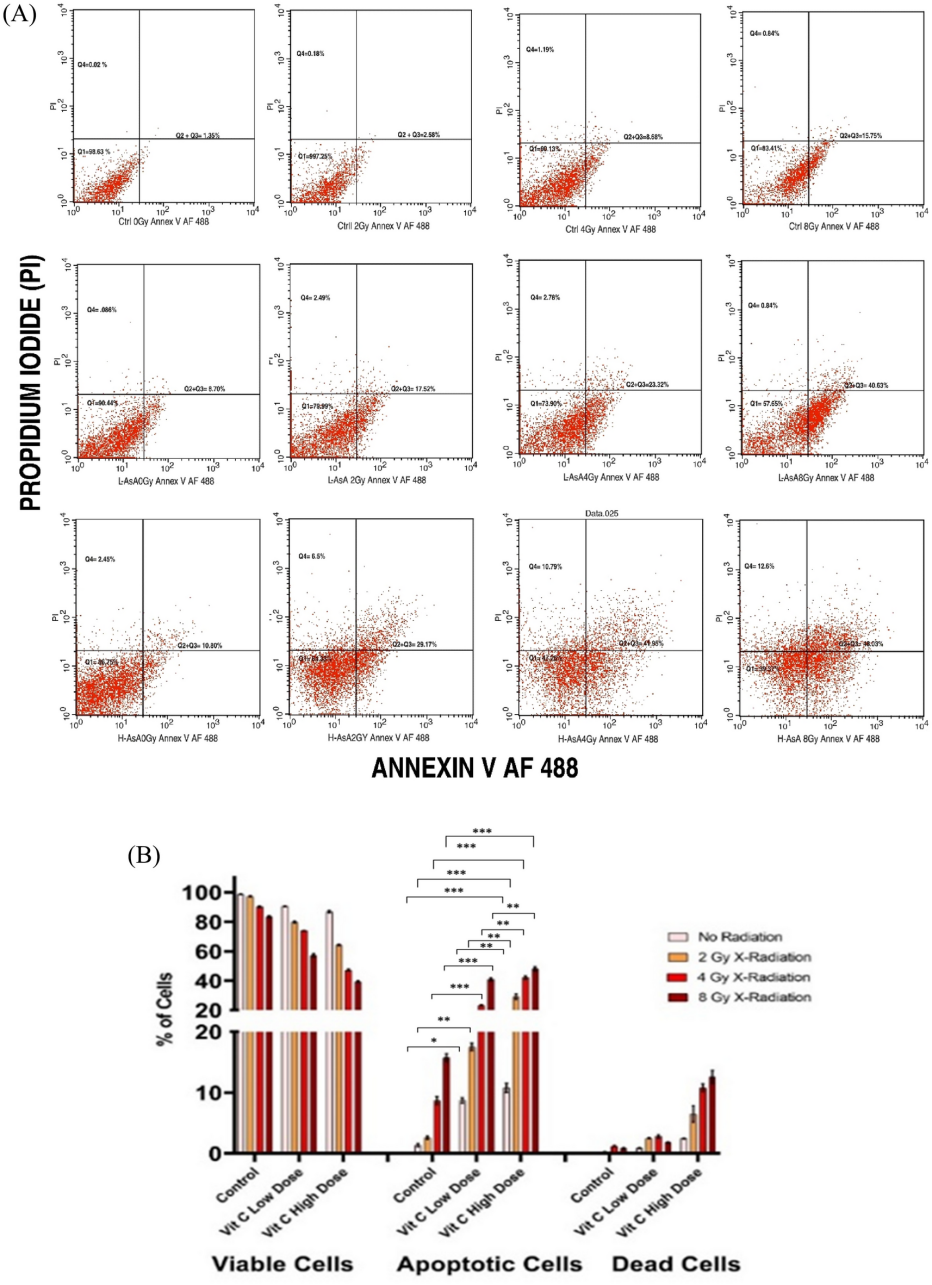

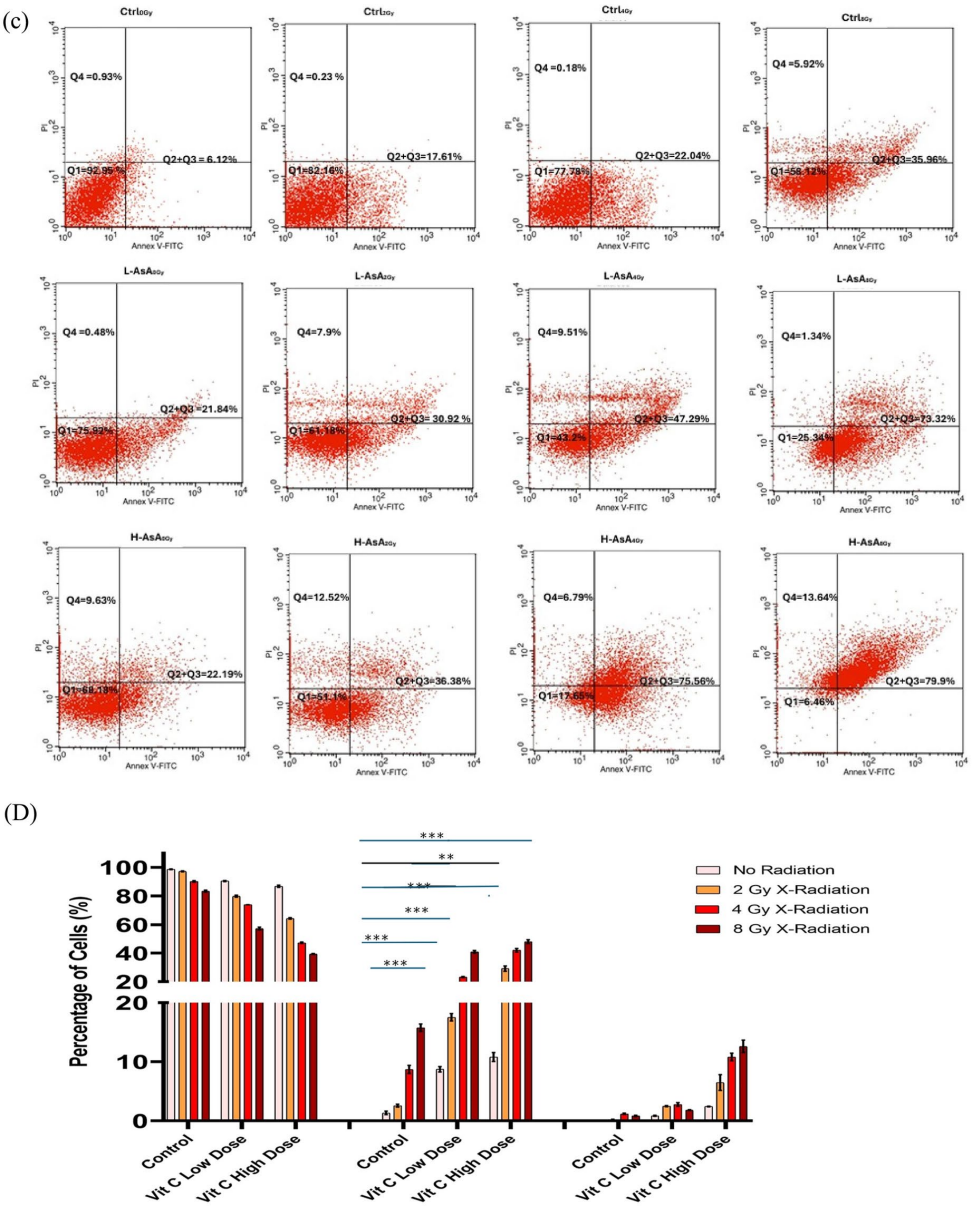
Flow cytometric analysis of Ascorbic Acid (AsA) and X-irradiation-induced apoptosis in THP-1 cells. (**A**) and (**C**) Representative flow cytometry plots using Annexin V-AF 488/PI staining for apoptosis for THP-1 & K562 cells, respectively. All cells were treated for 24 h and then stained with Annexin V-AF 488 and PI for flow cytometric analysis. Quadrant Q1 represents the percentage of viable cells. Quadrants Q2 and Q3 represent the percentages of cells in the early and late apoptotic phases, respectively. Quadrant Q4 represents the percentage of dead cells. (**B**) and (**D**) The bar chart shows the percentage of THP-1%K562 cell, respectively in each apoptotic stage. Data are presented as the mean ± standard deviation. Statistical significance is indicated as follows: ***p < 0.001, **p < 0.01, *p < 0.05.

Both AsA and X-radiation induced cellular apoptosis in all cell lines, with cells transitioning from the viable to the early apoptotic phase, as shown in Fig. 3A,C.

The percentage of apoptotic THP-1 cells is illustrated in Fig. 3B. The apoptosis rates in the L-AsA groups with 0 Gy, 2 Gy, 4 Gy, and 8 Gy X-irradiation concentrations(8.70 ± 0.47, 17.51 ± 0.62, 23.32 ± 0.38, 40.86 ± 0.95; respectively) were higher than that of Ctrl_0Gy_, Ctrl_2Gy_, Ctrl_4Gy_, and Ctrl_8Gy_ groups (1.35 ± 0.29, 2.57 ± 0.24, 8.68 ± 0.68, 15.75 ± 0.64; respectively) were significantly higher compared to the Ctrl groups (0 Gy, 2 Gy, 4 Gy, and 8 Gy X-irradiation) (1.35 ± 0.29, 2.57 ± 0.24, 8.68 ± 0.68, and 15.75 ± 0.64, respectively) with statistical significance (p < 0.05). Additionally, the apoptosis rates in the H-AsA groups with 0 Gy, 2 Gy, 4 Gy, and 8 Gy X-irradiation (10.78 ± 0.76, 29.13 ± 1.85, 41.95 ± 1.27, and 48.04 ± 1.44, respectively) were significantly higher than those in the corresponding Ctrl groups (1.35 ± 0.29%, 2.57 ± 0.24%, 8.68 ± 0.68%, and 15.75 ± 0.64%, respectively).

Furthermore, the proportions of early and late apoptotic K562 control group cells (Q_2_ and Q_3_ phases) were significantly higher for Ctrl_4Gy_ and Ctrl_8Gy_ as compared to both Ctrl_0Gy_ and Ctrl_2Gy_ groups (i.e., 22.04 ± 0.56 and 35.96 ± 1.16% vs. 6.12 ± 0.57 and 17.61 ± 0.58%, respectively, *P* < 0.05 for all associations). Compared to the control group, the number of apoptotic cells in the Q_2_ and Q_3_ phases increased further at both low and high concentrations of AsA of 4 and 8 mg/ml, regardless of the X-irradiation dose (Fig. 3C). Specifically, the percentages of apoptotic cells for L-AsA_0Gy_, L-AsA_2Gy_, L-AsA_4Gy_, and L-AsA_8Gy_ groups (i.e., 21.84 ± 0.57, 30.92 ± 0.61, 47.29 ± 1.33, and 73.32 ± 1.71%, respectively) were higher than those for Ctrl_0Gy_, Ctrl_2Gy_, Ctrl_4Gy_, and Ctrl_8Gy_ groups (i.e., 6.12 ± 0.57, 17.61 ± 0.58, 22.04 ± 0.56 and 35.96 ± 1.16%, respectively, *P* < 0.05 for all associations). Moreover, the percentage of apoptotic cells for H-AsA_0Gy_, H-AsA_2Gy_, H-AsA_4Gy_, and H-AsA_8Gy_ groups (i.e., 22.19± 0.56, 36.38 ± 1.25, 75.56 ± 1.14, and 79.9 ± 1.71%, respectively) were significantly higher compared with those for both the control and low-dose AsA groups (*P* < 0.05 for all associations).

Our study further highlights the differential impact of high-dose (H-AsA) versus low-dose (L-AsA) Ascorbic Acid on inducing apoptosis in THP-1 cells, particularly when combined with X-irradiation. Apoptosis rates in the L-AsA groups increased significantly with higher doses of X-irradiation compared to the baseline L-AsA_0Gy_ group.

L-AsA_2Gy_: Increased by 2.6-fold compared to L-AsA_0Gy,_ L-AsA_4Gy_: Increased by 3.8-fold compared to L-AsA_0Gy,_ L-AsA_8Gy_: Increased by 4.5-fold compared to L-AsA_0Gy_

These results underscore the potent apoptotic effects of high-dose AsA combined with X-radiation on THP-1cells. Both L-AsA and H-AsA demonstrated dose-dependent increases in apoptosis, but the H-AsA was more effective at inducing cell death at each level of X-radiation. AsA and/or X-radiation decreases the survival fraction of THP-1 in a dose-dependent manner.

### Colony formation assay

**Fig. 4:**
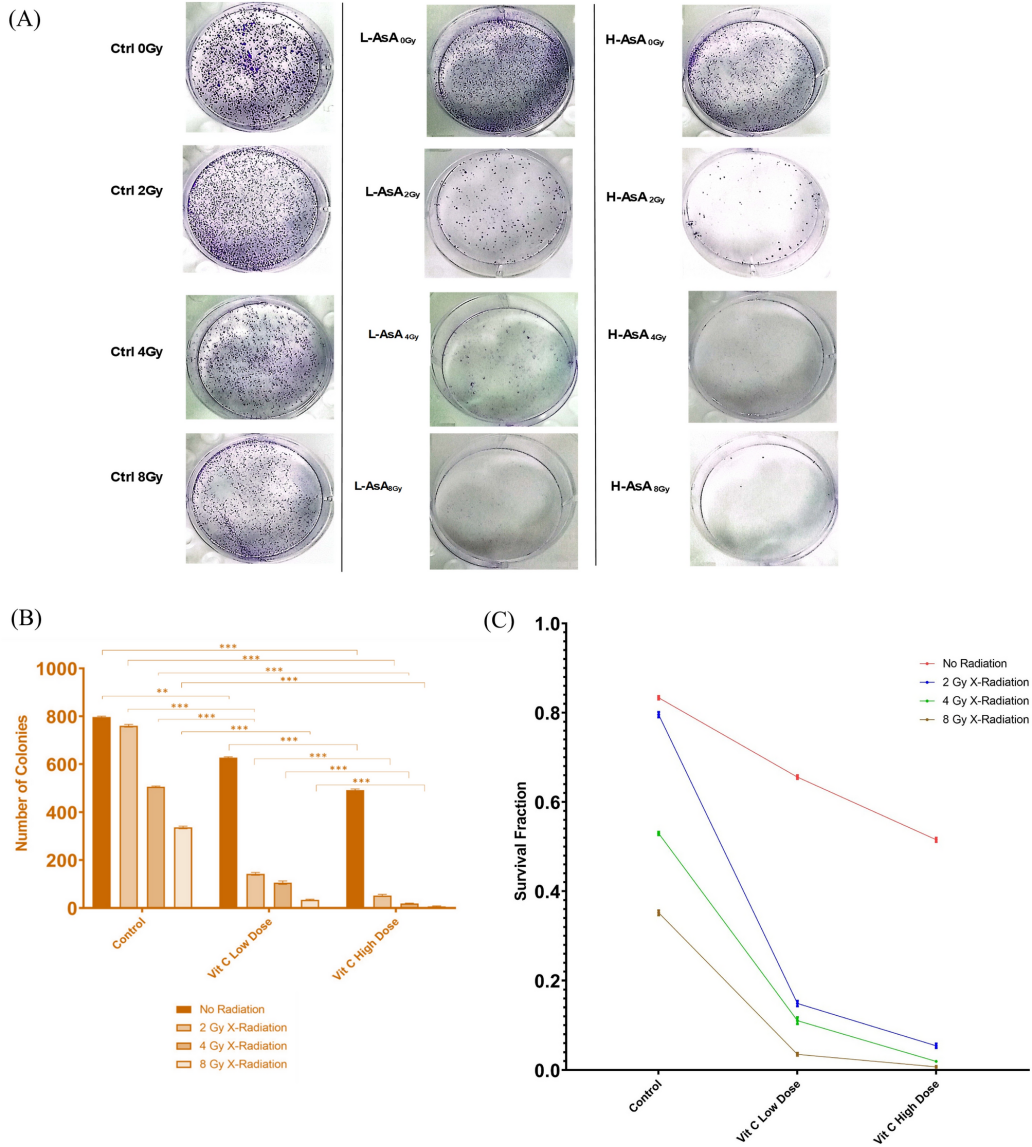
Effect of AsA and X- radiation on THP-1 cells lines ‘colonies formation. (**a**) Colony formation images, stained with 0.01% crystal violet for 1 h, only the cells with a diameter greater than 50 μM that were counted. (**b**) The bar chart of the number of the of THP-1 colonies. (**c**) The survival fraction of the THP-1 colonies normalized to the plating efficiency of the untreated control cells.

The clonogenic assay is the method of choice to determine cell reproductive death after treatment with ionizing radiation but can also be used to determine the effectiveness of other cytotoxic agents. Here, the treatments involve different doses of AsA (2.5 & 5 µg/mL) and/or X- irradiation (2 Gy, 4 Gy, 8 Gy), along with control groups. Ctrl 0 Gy shows a high number of colonies, indicating normal cell proliferation. As the X-radiation doses increase (2 Gy, 4 Gy, and 8 Gy), the number of colonies increases, demonstrating the dose-dependent cytotoxic effect of X- irradiation. In addition, the colonies are still detectable in the L-AsA 0 Gy group, but they are less than in the control without AsA, implying that the low AsA treatment alone causes some cytotoxicity. As X-radiation doses increase (2 Gy, 4 Gy, and 8 Gy), colony numbers gradually fall, particularly at 8 Gy, where colonies are almost nonexistent. Furthermore, the number of colonies in H-AsA 0 Gy is much lower than in the control group, demonstrating that high AsA concentration alone has a stronger cytotoxic effect. With increasing radiation doses (2 Gy, 4 Gy, and 8 Gy), the colonies nearly completely diminish and die, particularly at 8 Gy, where no visible colonies remain. This shows a substantial cytotoxic interaction between high AsA concentrations and X-rays. These findings confirm the synergistic effect between AsA and X-radiation, which leads to increased cell death in a dose dependent manner (Fig. 4a,b)>

The cell survival curve (SF) was made to assess the cytotoxic effects of AsA and/or X-irradiation on THP-1 cells. The SF and plating efficiency (PE) were estimated as described in the methodology. The PE was calculated to be 80%. The SF is the number of colonies that emerge following treatment, normalized to the PE of the untreated control. The SF for the control group was (99% ± 0.46%). Cells treated with 2 Gy (95.44% ± 0.72%), 4 Gy (63.52% ± 0.45%), and 8 Gy (42.21% ± 0.66%) X-radiation alone without AsA had considerably lower SF than the control group (p < 0.0001). The SF for cells treated with 2.5 and 5 µg/ml AsA without X-rays was 78.68% ± 0.50% and (61.83% ± 0.57%), respectively. In the L-AsA group, the SF was considerably lower than in the control group. Increasing X-radiation doses (L-AsA2Gy, L-AsA 4 Gy, and L-AsA 8 Gy) resulted in a reduction in survival (17.85% ± 0.73%, 13.24% ± 0.91%, and 4.23% ± 0.37%, respectively), with the lowest SF at the highest X-radiation dose (8 Gy). Additionally, the H-AsA group shows the most significant decline in the SF. The survival rate at H-AsA 0 Gy is already much lower than that of the control group. As radiation dose increases (H-AsA2Gy, H-AsA 4 Gy, and H-AsA 8 Gy) (6.52% ± 0.58%), (2.32% ± 0.26%), (0.87% ± 0.20%), the SF rapidly decreases, approaching zero at 8 Gy X-radiation, indicating nearly total cell death (Fig. 4c).

### The magical role of ascorbic acid in thwarting the proliferation of THP-1 cells

**Fig. 5:**
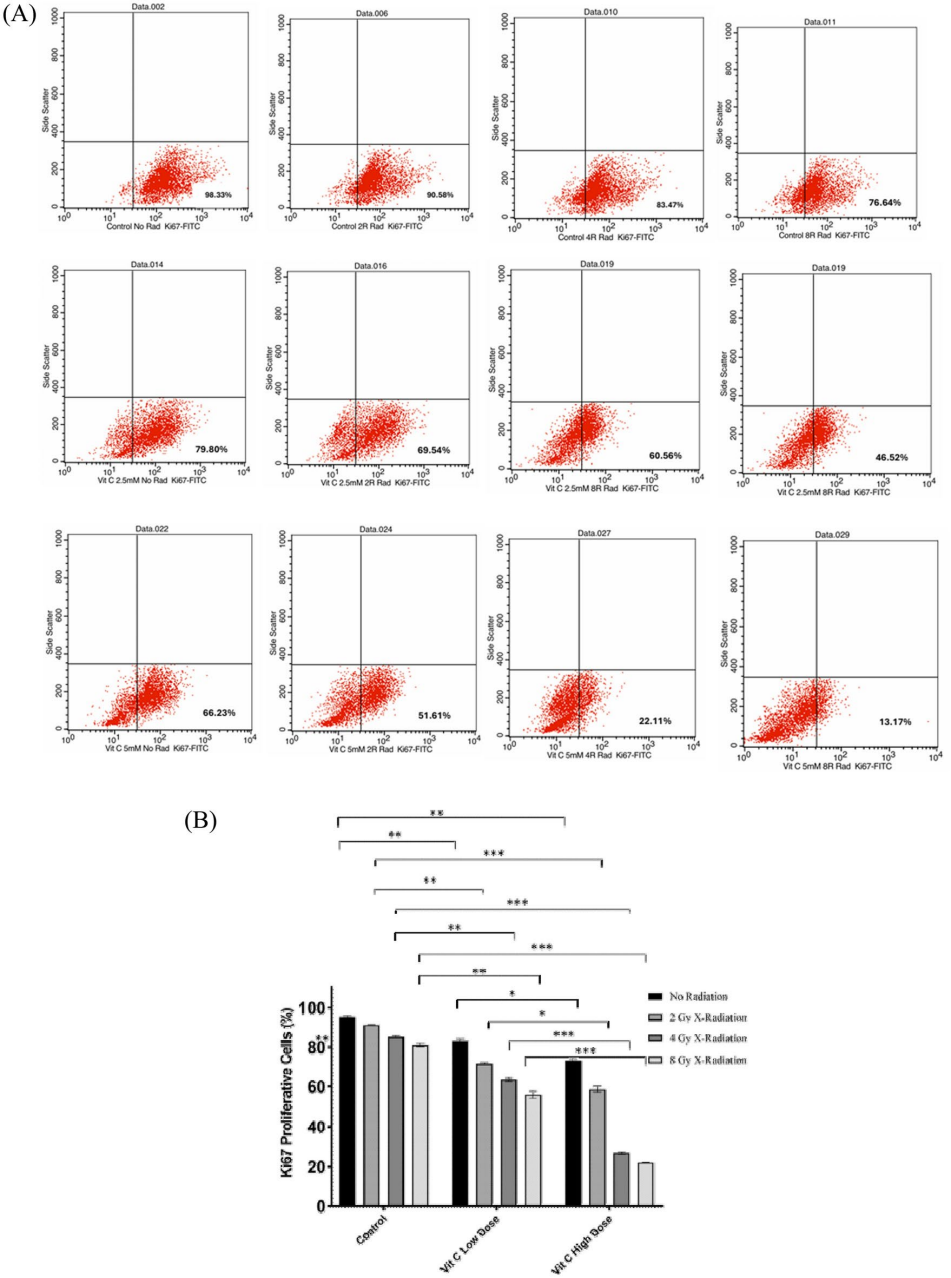
ki-67 protein in THP-1 Cells, after 24 h of treatment with different doses of AsA and X-radiation. (**A**) The dot plot analysis shows Ki-67 expression in THP-1 cells after treatment with AsA and/or radiation. The data illustrate the proportion of Ki-67-positive cells, reflecting cellular proliferation. Variation in Ki-67 expression across different treatment groups suggests changes in proliferative activity, potentially indicating treatment-induced modulation of cell cycle dynamics. Cell proliferation decreases when the dose of AsA increases like the proliferation of the H-AsA 4 Gy group was 22.11% compared with L-AsA or control groups. (**B**) The bar chart shows the percentage of Ki-67 levels. Data are presented as Mean ± Standard deviation. *P*-value < 0.05 that indicated by ***: 0.001, **: 0.01, *: 0.05.

THP-1 cells were exposed to various doses of AsA and X-radiation, and the changes in Ki67 expression were evaluated after 24 h, as shown in Fig. 5. The study revealed that Ki67 expression was lower in the L-AsA_0Gy_, L-AsA_2Gy_, L-AsA_4Gy_, and L-AsA_8Gy_ groups (82.93 ± 1.31, 71.79 ± 0.52, 63.37 ± 1.01, and 55.98 ± 1.88, respectively) compared to the Ctrl_0Gy_, Ctrl_2Gy_, Ctrl_4Gy_, and Ctrl_8Gy_ groups (95.27 ± 0.56, 90.81 ± 0.03, 85.13 ± 0.45, and 80.93 ± 0.82 respectively) with statistical significance (p < 0.05). Additionally, the proliferation rate in the H-AsA_0Gy_, H-AsA_2Gy_, H-AsA_4Gy_, and H-AsA_8Gy_ groups (73.22 ± 1.11, 58.58 ± 1.54, 26.56 ± 0.62, and 21.94 ± 0.15, respectively) was significantly reduced compared to the Ctrl_0Gy_, Ctrl_2Gy_, Ctrl_4Gy_, and Ctrl_8Gy_ groups (95.27 ± 0.56, 90.81 ± 0.03, 85.13 ± 0.45, and 80.93 ± 0.82, respectively).

Furthermore, the proliferation rate in the H-AsA_0Gy_, H-AsA_2Gy_, H-AsA_4Gy_, and H-AsA_8Gy_ groups was markedly lower compared to the L-AsA_0Gy_, L-AsA_2Gy_, L-AsA_4Gy_, and L-AsA_8Gy_ groups. Specifically, the proliferation rates in H-AsA_0Gy_, H-AsA_2Gy_, H-AsA_4Gy_, and H-AsA_8Gy_ (73.22 ± 1.11, 58.58 ± 1.54, 26.56 ± 0.62, and 21.94 ± 0.15, respectively) were reduced compared to L-AsA_0Gy_, L-AsA_2Gy_, L-AsA_4Gy_, and L-AsA_8Gy_ (82.93 ± 1.31, 71.79 ± 0.52, 63.37 ± 1.01, and 55.98 ± 1.88, respectively). Statistically, the proliferation in the L-AsA_2Gy_, L-AsA_4Gy_, and L-AsA_8Gy_ groups decreased by 1.2-fold, 1.3-fold, and 1.5-fold, respectively, compared to the L-AsA_0Gy_ group. Similarly, the percentage of Ki67 protein in cells was reduced in H-AsA_2Gy_, H-AsA_4Gy_, and H-AsA_8Gy_ by 1.2-fold, 2.8-fold, and 3.3-fold, respectively, compared to H-AsA_0Gy_.

### The effect of AsA with X-irradiation on THP-1 cells cell cycle

**Fig. 6:**
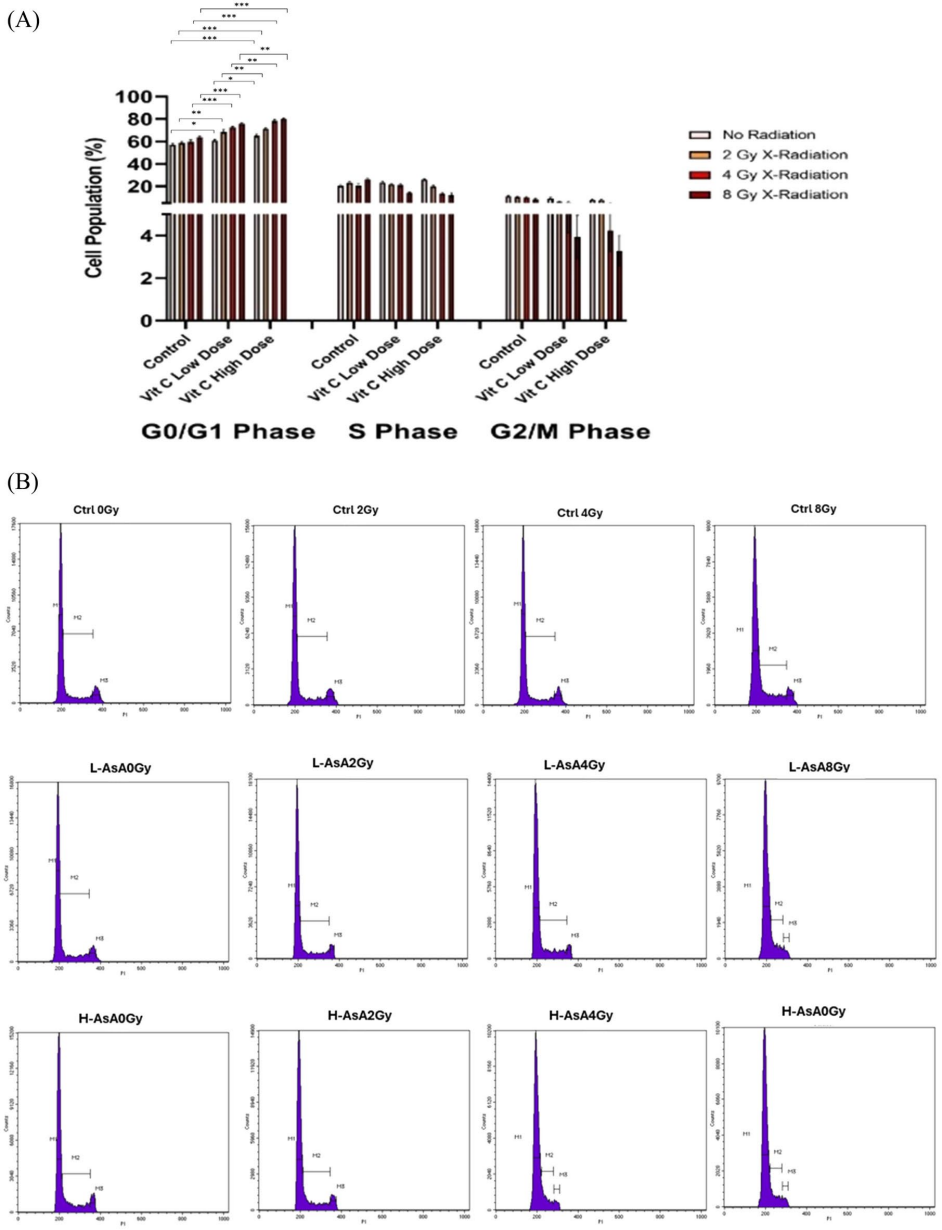
The progression of cell cycle for THP-1 cells after 24-h treatment with different doses of AsA and X-radiation. (**A**) The bar chart of THP-1 cells illustrated all phases of cell cycle after the treatment. Data are presented as Mean ± Standard deviation. P-value < 0.05, That indicated by ***: 0.001, **: 0.01, *: 0.05. As radiation dose increases, the percentage of cells in the S phase decreases, suggesting a significant inhibition of DNA synthesis. This decline implies that fewer cells are progressing through the replication phase, reinforcing the impact of G0/G1 arrest, the proportion of cells in the G2/M phase decreases indicating that fewer cells are reaching mitosis. (**B**) Cell cycle analysis of THP-1 cells following treatment with AsA andradiation. the histograms demonstrated the distribution of cells across different phases (G1, S, G2/M) the data suggest alterations in cell cycle progression depending on the dose of AsA and radiation exposure, highlighting a potential synergistic effect on cell cycle arrest.

The study reveals that combining AsA with X-radiation induces cell cycle arrest in THP-1 cells, particularly leading to an increase in the proportion of cells in the G0/G1 phase. This was analyzed after 24 hours of treatment using flow cytometry, as shown in Fig. 6. Our findings showed that THP-1 cells in the L-AsA groups showed a significantly higher percentage of cells arrested in the G0/G1 phase compared to their respective control groups.

(60.76 ± 1, 57, 68.74 ± 1.84, 72.41 ± 1.06, 75.69 ± 0.90; respectively), compared to Ctrl_0Gy_, Ctrl_2Gy_, Ctrl_4Gy_, and Ctrl_8Gy_ groups (57.10 ± 1.05, 58.65 ± 1.25, 59.74 ± 1.62, 63.83 ± 1.1963.83 ± 1.19 respectively) with a statistical significance (p < 0.05). Furthermore, the percentage of cell populations in H-AsA_0Gy_, H-AsA_2Gy_, H-AsA_4Gy_, and H-AsA_8Gy_, groups (65.19 ± 1.59, 71.29 ± 1.05, 78.42 ± 1.56, 78.42 ± 1.56; respectively) were higher than Ctrl_0Gy_, Ctrl_2Gy_, Ctrl_4Gy_, and Ctrl_8Gy_ groups (57.10 ± 1.05, 58.65 ± 1.25, 59.74 ± 1.62, 63.83 ± 1.19,63.83 ± 1.19; respectively).

Additionally, the cell population in G0/G1 phase of H-AsA_0Gy_, H-AsA_2Gy_, H-AsA_4Gy_, and H-AsA_8Gy_ groups (65.19 ± 1.59, 71.29 ± 1.05, 78.42 ± 1.56, 78.42 ± 1.56; respectively) were markedly increased compared with L-AsA_0Gy_, L-AsA_2Gy_, L-AsA_4Gy_, and L-AsA_8Gy_ groups (60.76 ± 1, 57, 68.74 ± 1.84, 72.41 ± 1.06, 75.69 ± 0.90; respectively).

These findings indicate that AsA, particularly in higher concentrations, effectively induces cell cycle arrest in the G0/G1 phase when combined with X-radiation. The impact is more pronounced in the H-AsA groups, showing a stronger capacity to halt cell cycle progression compared to both the L-AsA and control groups. This arrest in the G0/G1 phase suggests a mechanism by which AsA enhances the effects of X-radiation, potentially contributing to reduced cell proliferation and increased apoptosis in THP-1 cells.

The combination of AsA and X-radiation not only induced cell cycle arrest in the G0/G1 phase but also resulted in a significant decrease in the S and G2/M phase cell populations, further indicating that AsA inhibits cell growth by preventing cell cycle progression beyond the G0/G1 phase, and also reduced the proportion of cells in the S and G2/M phases, indicating that AsA combined with X-radiation effectively halts cell cycle progression, limiting cell growth by causing arrest at the G0/G1 phase.

The percentage of cells in the G0/G1 phase was significantly higher in the H-AsA groups compared to the L-AsA groups across all X-radiation doses, in addition to our previous explanations, the cell populations for all L-AsA groups in the G0/G1 phase increased as X-ray doses increased: L-AsA_2Gy_: 1.13-fold increase compared to L-AsA_0Gy,_ L-AsA_4Gy_: 1.19-fold increase compared to L-AsA_0Gy,_ L-AsA_8Gy_: 1.25-fold increase compared to L-AsA_0Gy_

Similarly, in the H-AsA groups, the G0/G1 phase cell populations showed a significant increase with increasing the X- ray dose.

H-AsA_2Gy_: 1.09-fold increase compared to H-AsA_0Gy,_ H-AsA_4Gy_: 1.20-fold increase compared to H-AsA_0Gy,_ H-AsA_8Gy_: 1.23-fold increase compared to H-AsA_0Gy_

These results demonstrate that AsA, especially at higher doses, combined with X-radiation, more effectively induces cell cycle arrest at the G0/G1 phase, preventing cells from progressing through the cell cycle. This inhibition of cell cycle progression is a critical mechanism by which AsA enhances the anti-proliferative effects of X-radiation in THP-1 cells.

### The association between AsA and X-irradiated THP-1 cells at the level of HIF-1

**Fig. 7:**
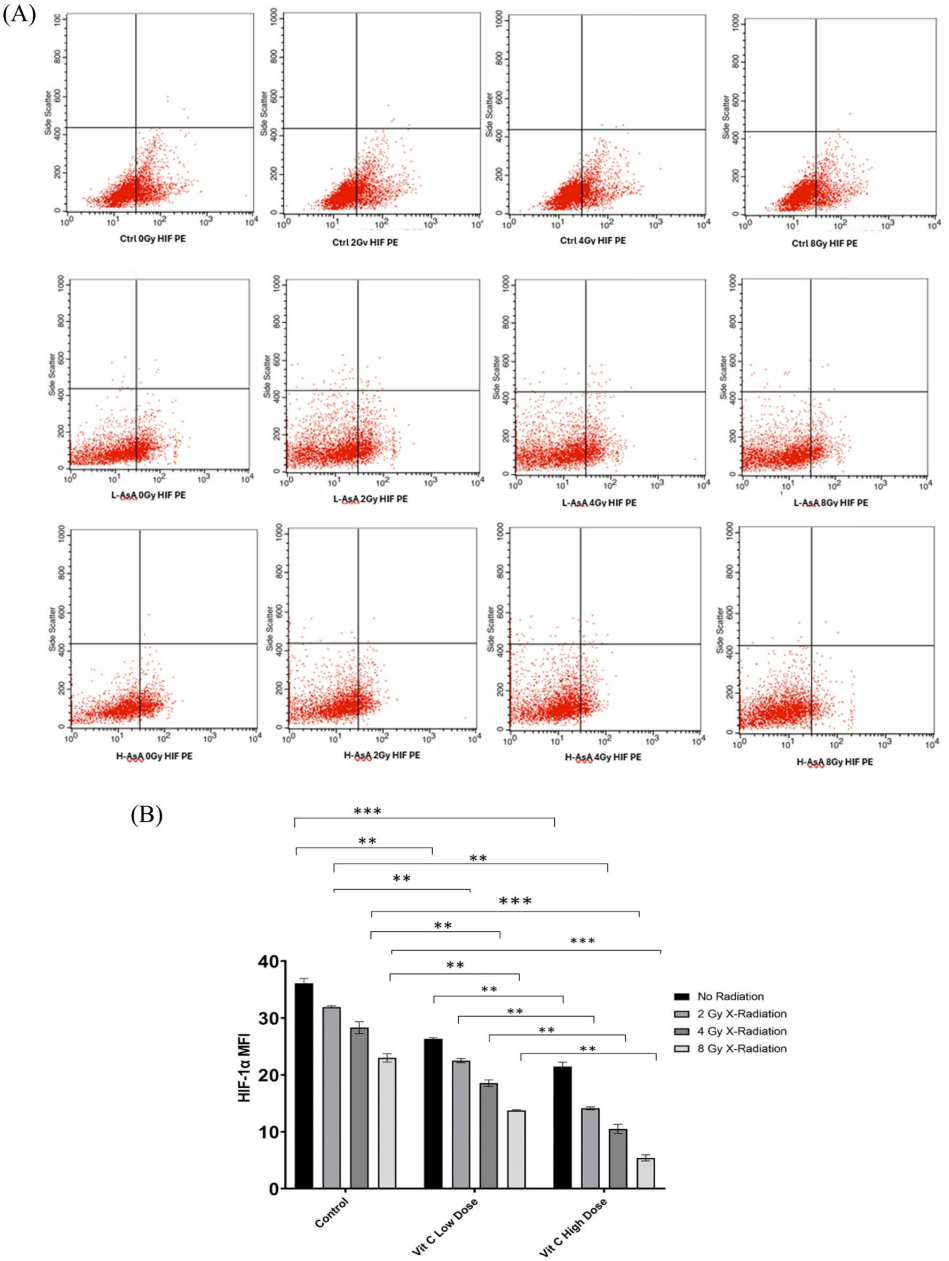
The percentage of HIF levels of THP-1 leukemic cell line after being treated by AsA and X-radiation. (**A**) The dot plot of treated THP-1 cells that showed the percentage of HIF stained with PE dye by flow cytometry analysis. (**B**) The bar chart indicates HIF -expression in THP-1 cells that were treated with AsA and X-radiation. Data are presented as Mean ± Standard deviation. P-value < 0.05. That represented by ***: 0.001, **: 0.01, * : 0.05.

Our results demonstrated a prominent decrease in HIF-1α level in LAsA_0Gy_, L-AsA_2Gy_, L-AsA_4Gy_, and L-AsA_8Gy_ groups (26.33 ± 0.19, 22.51 ± 0.38, 18.53 ± 0.57, 13.75 ± 0.10; respectively) compared to of Ctrl_0Gy_, Ctrl_2Gy_, Ctrl_4Gy_, and Ctrl_8Gy_ groups (36.11 ± 0.82, 31.92 ± 0.18, 28.31 ± 1.06, 22.99 ± 0.72; respectively) with a statistical significance (p < 0.05) as shown in Fig. 7.

Reduction in HIF-1α levels in the H-AsA groups compared to the control groups, indicating a stronger inhibitory effect of high-dose AsA.

H-AsA_0Gy_: 21.5 ± 0.76 vs. Ctrl_0Gy_: 36.11 ± 0.82, H-AsA_2Gy_: 14.11 ± 0.26 vs. Ctrl_2Gy_: 31.92 ± 0.18

H-AsA_4Gy_: 10.49 ± 0.82 vs. Ctrl_4Gy_: 28.31 ± 1.06, H-AsA_8Gy_: 5.39 ± 0.54 vs. Ctrl_8Gy:_ 22.99 ± 0.72.

Both low L-AsA and high H-AsA combined with X-radiation significantly decreased HIF-1α levels in THP-1 cells in a dose-dependent manner.

The H-AsA groups showed a more pronounced reduction in HIF-1α levels compared to the L-AsA groups, highlighting the stronger effect of higher AsA concentrations in inhibiting HIF-1α, which is crucial for hypoxia regulation and cancer cell survival.

Statistically, in comparison with the L-AsA_0Gy_ group, HIF1-α level in L-AsA_2Gy_, L-AsA_4Gy_, and L-AsA_8Gy_ groups was prominently decreased by (1.17, 1.4, 1.9-fold; respectively). The level of HIF1-α level in THP-1 cells was also significantly reduced in H-AsA_2Gy_, H-AsA_4Gy_, and H-AsA_8Gy_ (1.5, 2, 3.9-fold; respectively) compared to H-AsA_0Gy_.

### Ascorbic acid increases the THP-1 cells ‘autophagia process

**Fig. 8:**
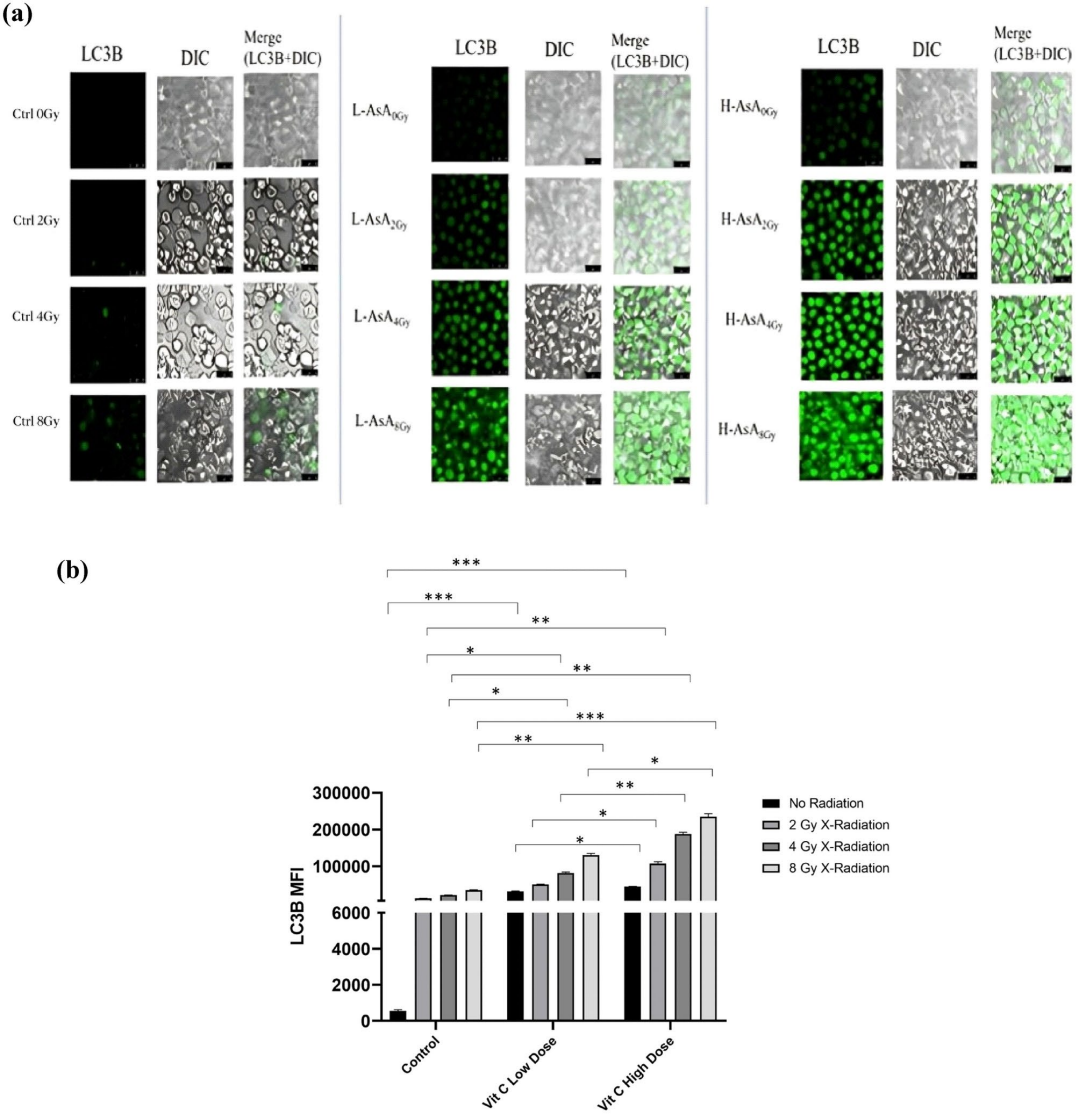
Effects of AsA and X- radiation on the autophagia behavior of THP-1 cells. (**a**) Autophagy was determined with immunofluorescence staining for LC3B by laser confocal microscope. (**b**). The bar chart indicates LC3B -expression in THP-1 cells that were treated with AsA and X-irradiation. Data are presented as mean ± standard deviation. p < 0.05, that indicates As ***: 0.001, **: 0.01, *: 0.05.

LC3B, an autophagic marker, is one of the first identified mammalian proteins that localizes to the autophagosome membrane^40^. This study used immunofluorescence to assess the distribution of LC3B, and the results were presented in Fig. 8a. Cells treated with H-AsA showed significantly higher intensity of LC3B, indicating increased autophagic activity as shown in Fig. 8b.

The intensity of LC3B in L-AsA groups with the varying radiation doses was:

L-AsA_0Gy_: 31,227.32 ± 1362.73, L-AsA_2Gy_: 50,123.35 ± 1419.55.

L-AsA_4Gy_: 81,804.47 ± 2281.94, L-AsA_8Gy:_ 130,185.73 ± 4812.57 was significantly higher compared to the Control groups with varying radiation doses (551.11 ± 65.94, 12,490.05 ± 70.29, 21,312.14 ± 228.74, and 34,925.11 ± 1137.85, respectively), with statistical significance (p < 0.05)

LC3B intensity in the L-AsA groups was significantly higher compared to the corresponding Control groups, with statistical significance (p < 0.05).

In summary, treatment with L-AsA led to increased autophagic activity, as evidenced by the elevated LC3B intensity, which was significantly higher compared to the control groups across various radiation doses.

The LC3B levels in the H-AsA_0Gy_, H-AsA_2Gy_, H-AsA_4Gy_, and H-AsA_8Gy_ groups (44,597.67 ± 838.08, 106,957.24 ± 5120.38, 187,561.65 ± 5079.08, and 1,234,828.04 ± 8299.33, respectively) were significantly higher compared to the Ctrl_0Gy_, Ctrl_2Gy_, Ctrl_4Gy_, and Ctrl_8Gy_ groups (551.11 ± 65.94, 12,490.05 ± 70.29, 21,312.14 ± 228.74, and 34,925.11 ± 1137.85, respectively).

Furthermore, the level of LC3B in the H-AsA_0Gy_, H-AsA_2Gy_, H-AsA_4Gy_, and H-AsA_8Gy_ groups was markedly increased compared to the L-AsA_0Gy_, L-AsA_2Gy_, L-AsA_4Gy_, and L-AsA_8Gy_ groups. Specifically, LC3B levels in the H-AsA_0Gy_, H-AsA_2Gy_, H-AsA_4Gy_, and H-AsA_8Gy_ groups (44,597.67 ± 838.08, 106,957.24 ± 5120.38, 187,561.65 ± 5079.08, and 1,234,828.04 ± 8299.33, respectively) were significantly higher than those in the L-AsA_0Gy_, L-AsA_2Gy_, L-AsA_4Gy_, and L-AsA_8Gy_ groups (31,227.32 ± 1362.73, 50,123.35 ± 1419.55, 81,804.47 ± 2281.94, and 130,185.73 ± 4812.57, respectively). Statistically, compared to the L-AsA_0Gy_ group, the LC3B marker in the L-AsA_2Gy_, L-AsA_4Gy_, and L-AsA_8Gy_ groups increased by 1.2-fold, 1.3-fold, and 1.5-fold, respectively. LC3B levels were also significantly augmented in the H-AsA_2Gy_, H-AsA_4Gy_, and H-AsA_8Gy_ groups by 1.2-fold, 2.8-fold, and 3.3-fold, respectively, compared to H-AsA_0Gy_.

LC3B levels in all H-AsA groups were significantly higher than those in the corresponding control groups, indicating that High Ascorbic Acid treatment leads to a substantial increase in LC3B expression, a marker for autophagy.

LC3B levels were also notably higher in the H-AsA groups compared to the corresponding L-AsA groups, H-AsA_0Gy_ vs. L-AsA_0Gy_: 44,597.67 vs. 31,227, H-AsA_2Gy_ vs. L-AsA_2Gy_: 106,957.24 vs. 50,123.35, H-AsA_4Gy_ vs. L-AsA_4Gy_: 187,561.65 vs. 81,804.47, H-AsA_8Gy_ vs. L-AsA_8Gy_: 1,234,828.04 vs. 130,185.7.

LC3B levels increased by 1.2-fold, 1.3-fold, and 1.5-fold in the L-AsA_2Gy_, L-AsA_4Gy_, and L-AsA_8Gy_ groups, respectively, compared to L-AsA_0Gy_.

LC3B levels increased by 1.2-fold, 2.8-fold, and 3.3-fold in the H-AsA_2Gy_, H-AsA_4Gy_, and H-AsA_8Gy_ groups, respectively, compared to H-AsA_0Gy_.

These results indicate that high AsA treatment, especially at higher radiation doses, significantly boosts LC3B levels more than Low Ascorbic Acid treatment, suggesting an enhanced autophagic response. The increasing fold changes in LC3B levels across both L-AsA and H-AsA groups as the radiation dose increases further underscore the synergistic effect of ascorbic acid andX- radiation in promoting autophagy.

### Measurement of Oxidative Stress Index (MDA/TAC Index)

**Fig. 9:**
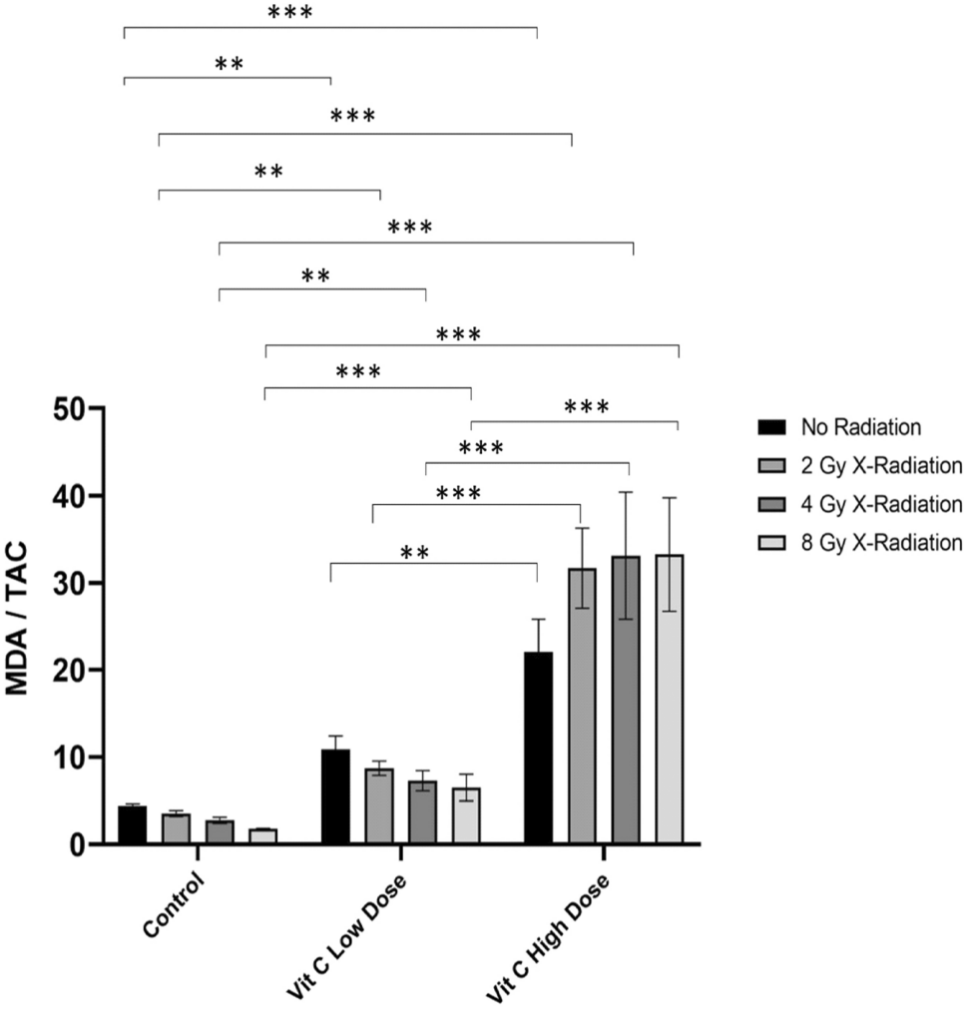
Effect of AsA and X-radiation on oxidative stress in THP-1 cells. Malondialdehyde/total antioxidant capacity (MDA/TAC) oxidative-stress index in THP-1 culture media collected after 1 day which treated with low and high doses of AsA followed by different doses of X-radiation (2, 4 & 8 Gy). The control group exhibited the lowest MDA/TAC levels, while treatment with AsA alone resulted in a mild increase, particularly at the high dose. X-radiation alone induced a dose-dependent rise in MDA/TAC, suggesting radiation-induced oxidative stress. The combination of AsA and X-radiation, the MDA/TAC ratio showed a synergistic effect, especially at the higher radiation doses (4 Gy and 8 Gy), with significantly increased oxidative stress compared to individual treatments. The highest oxidative stress levels were observed in the group treated with high-dose AsA and 8 Gy X-radiation. Results were presented as mean ± standard deviation, That represented by ***: 0.001, **: 0.01, *: 0.05.

The results of our experiment analyzed the MDA/TAC. (MDA), a product of free-radical-induced oxidation of polyunsaturated fatty acids, to total antioxidant capacity (TAC) across different groups exposed to various conditions were shown in Fig. 9. The MDA/TAC ratio serves as an oxidative stress marker, where higher values indicate increased lipid peroxidation and reduced antioxidant defense.

The comparison between L-AsA Groups and Ctrl Groups explain that the MDA/TAC ratio was significantly higher in the L-AsA groups (L-AsA_0Gy_, L-AsA_2Gy_, L-AsA_4Gy_, L-AsA_8Gy_) compared to the Ctrl groups (Ctrl_0Gy_, Ctrl_2Gy_, Ctrl_4Gy_, Ctrl_8Gy_).

The reported values for the L-AsA groups ranged from 6.5 to 10.91, while the values for the Ctrl groups ranged from 1.79 to 4.43, indicating a statistically significant increase (p < 0.05), although the comparison Between H-AsA Groups and Ctrl Groups showed that the MDA/TAC ratio was much higher in the H-AsA groups (H-AsA_0Gy_, H-AsA_2Gy_, H-AsA_4Gy_, H-AsA_8Gy_) compared to the corresponding Ctrl groups.

The H-AsA group’s MDA/TAC values ranged from 22.07 to 33.25, which are markedly higher than the control values.

Finally, when we compare between H-AsA and L-AsA Groups the MDA/TAC ratio in the H-AsA groups was significantly higher than in the L-AsA groups.

Specifically, the H-AsA groups showed a 1.2- to 1.5-fold increase compared to the L-AsA_0Gy_ group. In the H-AsA_2Gy_, H-AsA_4Gy_, and H-_AsA8Gy_ groups, the MDA/TAC ratio increased by 1.2-fold, 2.8-fold, and 3.3-fold, respectively, compared to the H-AsA_0Gy_ group.

These results suggest that both L-AsA and H-AsA treatments elevated oxidative stress as indicated by the increased MDA/TAC ratio compared to the control groups, with a more pronounced effect in the H-AsA groups. Additionally, increasing radiation doses seem to further augment the oxidative stress levels, especially in the H-AsA group.

## Discussion

Despite significant advances in therapy, leukemia remains a challenging and often incurable disease^41^. Chemotherapy and radiation continue to be the mainstays of treatment^42^. Ionizing radiation generates reactive oxygen species (ROS), including hydroxyl radicals (·OH), which can cause substantial cellular damage. These radicals react immediately with nearby molecules, including DNA, leading to radiation-induced cell damage^43,44^. Ascorbic acid (AsA) is known to influence the efficacy of radiation treatment in leukemia. High doses of AsA generally exhibit cytotoxic effects, while lower doses can protect cells from oxidative stress^45^. To validate the anticancer effect of AsA, its impact was evaluated on multiple cell lines, including THP-1 and K562.

Apoptosis, programmed cell death, is crucial for maintaining tissue homeostasis, development, and immune function. It eliminates damaged or abnormal cells without causing inflammation or harm to the surrounding tissues^46^. Dysregulation of apoptosis can contribute to cancer development by allowing damaged cells to survive and proliferate uncontrollably^47^. Our study found that higher concentrations of AsA led to a decrease in viable cells and an increase in apoptotic cells, particularly evident in the H-AsA_0Gy_ group compared to the L-AsA_0Gy_ group. Shinozaki et al. observed similar effects in HL60 cells, where increasing AsA concentrations decreased cell viability but also reduced DNA fragmentation, a marker of apoptosis, at concentrations above 5 mM^45^. Our results align with these findings, showing that high-dose AsA (5 μg/ml) significantly increased the percentage of apoptotic cells compared to a lower dose (2.5 μg/ml) on THP-1 cells, and also in K562 cells, high dose of AsA (8 μg/ml) given more proportions of apoptosis than low dose (4 μg/ml). Additionally, high-dose X-radiation (8 Gy) induced more apoptosis than lower doses (2 or 4 Gy), consistent with Shinozaki’s observations that apoptosis was a major cause of cell death in HL60 cells exposed to up to 10 Gy of radiation^45^.

Our study demonstrated that AsA enhances the cytotoxic effect of X-radiation. For example, the H-AsA_2Gy_ group showed a higher percentage of apoptotic cells than the Ctrl_2Gy_ group, which received X-radiation alone. This finding supports the work of^48^, which indicated that combining AsA (≥ 1 mM) with X-ray radiation reduced cell survival more than X-radiation alone. Our data also showed that AsA’s effect was concentration-dependent, with the H-AsA_2Gy_ group showing a better outcome compared to the Ctrl_8Gy_ group.

Interestingly, a higher percentage of early apoptosis was observed in cells treated with high concentrations of AsA (5 μg/ml) and exposed to 8 Gy X-radiation on THP-1 cells compared to the untreated group. The percentage of viable cells in the Ctrl8Gy group (83.43%) was significantly higher than in the H-AsA_8Gy_ group (39.37%). This indicates that AsA, when combined with radiation, induces significant apoptosis in THP-1 cells. The percentage of apoptotic cells was markedly higher in the L-AsA_2Gy_ group (17.5%) compared to the Ctrl2Gy group (2.6%), highlighting AsA’s role in promoting apoptosis.

The clonogenic assay is a fundamental tool to quantify dose-dependent changes in cells’ proliferative capacities after irradiation and hence has been an indispensable assay in radiobiology for the last decades^49^. For reasons of feasibility and interpretability, this assay reduces the complex growth behavior of cell colonies to a scalar readout, i.e. a dose-dependent survival rate^50^.

We performed survival assays to assess the effects of AsA combined with X-radiation on the colony-forming ability of the THP-1 monocytic cell line. The results indicate that while AsA has no effect on normal cells, it enhances the efficacy of X-radiation in THP-1 cells, significantly affecting colony formation and cell viability. This aligns with findings by Domenico et al*.,* which demonstrate that leukemic and, especially, myeloid cell lines are highly sensitive to the cytotoxic effects of pharmacologic doses of AsA, whereas normal CD34 cells remain unaffected^51^.

X-radiation affected the survival rate of THP-1 cell lines, consistent with findings by Fitzgerald M.D. et al*.*^52^, who demonstrated that conventional dose rates of ionizing radiation affect the clonogenic survival of leukemia and lymphoma cell lines.

AsA also exhibited an inhibitory effect on THP-1 cell proliferation, as evidenced by Ki-67 staining. Ki-67, a marker of cell proliferation, is expressed during active phases of the cell cycle (G1, S, G2, and M), but not during the resting phase (G0)^53^. Our results showed that high-dose AsA (5 μg/ml) led to the lowest proliferation rate in THP-1 cells exposed to 8 Gy radiation. This finding is consistent with^54^, which reported that high doses of AsA (8 and 20 mmol/L) suppressed cell proliferation in HL-60 and U937 cells. Our data confirmed that increasing AsA concentration decreased proliferation while increasing apoptosis.

X-radiation with AsA caused a notable reduction in THP-1 cell development by inducing cell cycle arrest in G0/G1 phases. The cell cycle data corroborated the reduction in Ki-67 levels. For example, the percentage of cells in the G0/G1 phase increased from 60.76% to 65.19% with AsA treatment, while cells in the G2/M phase decreased. This finding aligns with Zhou et al., who observed that AsA inhibits the G0/G1 phase of the cell cycle in various cancer cells^55^.

Hypoxia is a significant barrier to effective tumor therapy because it makes tumors more resistant to radiotherapy and chemotherapy^56,57^. Tumor development is related to hypoxia; hence the cells must have tools to live under hypoxic conditions^58^. Our study found that HIF-1α, a protein induced by hypoxia and X-radiation, was significantly suppressed in the H-AsA_8Gy_ group. This suppression of HIF-1α was associated with decreased cell viability in THP-1 cells following X-radiation.

The MDA/TAC index, which measures oxidative stress by comparing MDA levels (a product of oxidative damage) to TAC levels (antioxidant capacity), showed an increased ratio at high AsA concentrations. The MDA/TAC ratio decreased approximately 18.5-fold in the H-AsA_8Gy_ group compared to the L-AsA8Gy group, indicating reduced oxidative stress. AsA’s antioxidant properties may buffer ROS generated by radiation, protecting normal tissues from damage^59^.

Radiation therapy is a common therapeutic cancer treatment that causes irreversible DNA damage by direct damage or the creation of oxidative radicals when interacting with water molecules. On the other hand, it can produce ROS and harm tumor cells as well as normal tissues^60^. Previous studies have shown that AsA can enhance the cytotoxic effects of radiation while protecting normal cells^61^.

Autophagy, a cellular process for degrading damaged organelles and proteins, is critical in leukemia. It can act as a survival mechanism or lead to cell death depending on its regulation^62^. LC3B, a marker for autophagy^63^ was significantly upregulated in AsA-treated THP-1 cells^64^. Excessive autophagy, as seen with high-dose AsA, can lead to autophagic cell death, characterized by increased autophagic vacuoles^65,66^. Our study found that AsA enhanced LC3B expression, especially at high doses (5 μg/ml), indicating increased autophagy.

Ionizing radiation promotes autophagy^67^, although X-radiation alone did not significantly affect LC3B levels, combining AsA with X-radiation showed improved results in LC3B expression in the H-AsA_8Gy_ group.

## Conclusion

Ascorbic acid demonstrates potential as an effective antitumor agent against leukemia. Our study provides insight into its cytotoxic effects and underlying mechanisms, suggesting that AsA, whether administered in high or low doses, could enhance the efficacy of X-radiation while mitigating its potential hazards. These findings support further exploration of AsA’s therapeutic use in conjunction with radiation therapy for leukemia.

## Recommendations

The observed radioprotective effects of vitamin C at the doses used in this study highlight its potential as a non-toxic, cost-effective, and readily available radioprotector. This opens new avenues for developing strategies to protect against the harmful effects of ionizing radiation, particularly in critical situations involving high radiation doses.

To further validate these findings, it is essential to conduct additional research at both molecular and cellular levels.

Future studies should have to expand experimental models by Utilizing a broader range of cell lines (K562) and experimental animal models to confirm the safety and efficacy of vitamin C in various biological contexts and to better understand its radioprotective mechanisms, also optimize the dosage and administration by investigating the optimal dosing regimens and administration routes for vitamin C to maximize its radioprotective effects while minimizing any potential side effects.

Assess the long-Term Effects of AsA by evaluating the long-term impacts of vitamin C on health and cell function to ensure that its radioprotective benefits do not come with adverse effects.

Explore synergistic effects by examining the potential for vitamin C to enhance the efficacy of other radioprotective agents or therapies, creating a synergistic approach to radiation protection.

Finally design and conduct clinical trials to assess the effectiveness of vitamin C in human populations, particularly in scenarios of radiation exposure, such as cancer treatments or accidental radiation exposure.

By addressing these research areas, we can better understand and potentially harness the benefits of vitamin C as a radioprotective agent, contributing to improved safety and efficacy in radiation-related contexts.

## Data Availability

All data available upon my request, the corresponding author.
